# Supplementary material for: BRI1 and BAK1 interact with G proteins and regulate sugar-responsive growth and development in Arabidopsis
Source: Nat Commun. 2018 Apr 18;9:1522. doi: 10.1038/s41467-018-03884-8 (PMC5906681; doi:10.1038/s41467-018-03884-8)
Supplement: Supplementary file 1 — Supplementary Information [file 41467_2018_3884_MOESM1_ESM.pdf]

Supplementary Information

BRI1 and BAK1 interact with G proteins and regulate sugar-responsive growth and development in *Arabidopsis*

Peng et al.

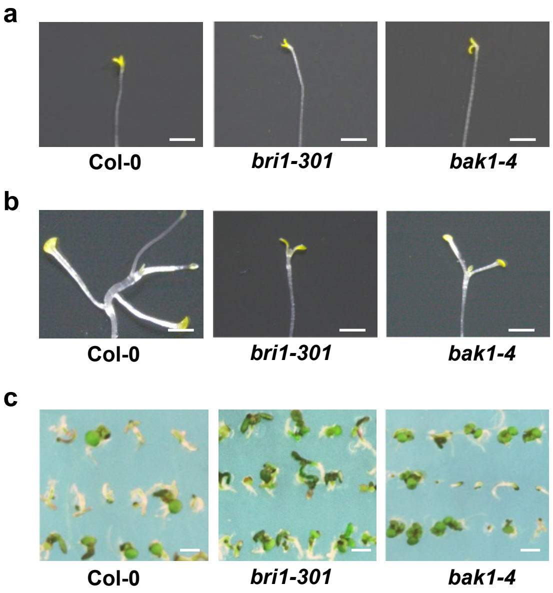

Supplementary Figure 1 | Phenotypes of BR-related mutants grown on medium with or without glucose
(a,b) Col-0, *bri1-301* and *bak1-4* seedlings were grown on MS medium with 1% mannitol (a) or 1% glucose (b) in the dark for 19 days. (Scale bars, 2 mm.)
(c) Col-0, *bri1-301* and *bak1-4* seedlings were grown on MS medium with 6% glucose in constant light for 9 days. (Scale bars, 10 mm.)

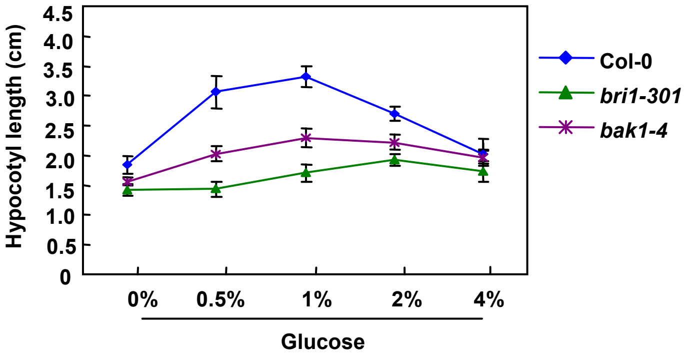

Supplementary Figure 2 | BRI1 and BAK1 are required for glucose-regulated hypocotyl elongation. Hypocotyl length of Col-0, *bri1-301* and *bak1-4* seedlings grown on medium with different concentrations of glucose in the dark for 17 days (n $\geqslant$ 64).

Values are given as mean $\pm$ standard deviation (SD).

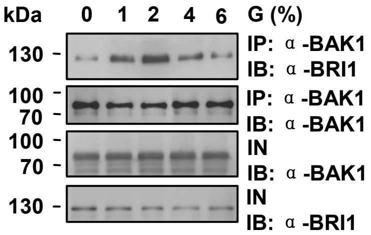

Supplementary Figure 3 | Glucose influences the interactions between BRI1 and BAK1.
Glucose influences the physical interactions between BRI1 and BAK1 in a concentration-dependent manner. Wild type seedlings were grown under light condition for 5 days, then incubated in darkness for 4 days, and treated with glucose as indicated for 6 hours. Total proteins were isolated and incubated with anti-BAK1 antibody for 30min, and then incubated with Protein G Magnetic Beads for 30min to immunoprecipitate BAK1. Immunoprecipitated proteins were detected with anti-BRI1 and anti-BAK1 antibodies, respectively. IN, input; IP, immunoprecipitation; IB, immunoblot; G, glucose.

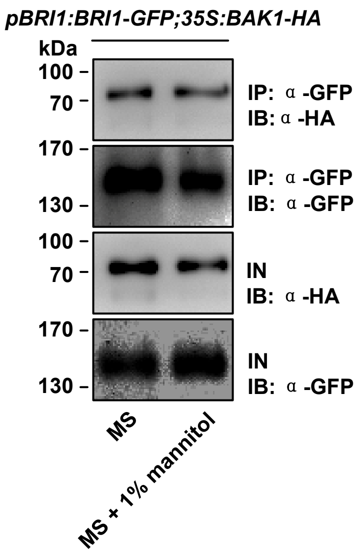

Supplementary Figure 4 | Mannitol does not affect the interaction between BRI1 and BAK1. *pBRI1:BRI1-GFP;35S:BAK1-HA* seedlings were grown under light condition for 5 days, incubated in darkness for 4 days, and then treated with 1% mannitol for 4 hours. Total proteins were isolated and incubated with green fluorescent protein (GFP)-Trap-A agarose beads to immunoprecipitate BRI1-GFP. Immunoprecipitated proteins were detected with anti-GFP and anti-HA antibodies, respectively. IN, input; IP, immunoprecipitation; IB, immunoblot.

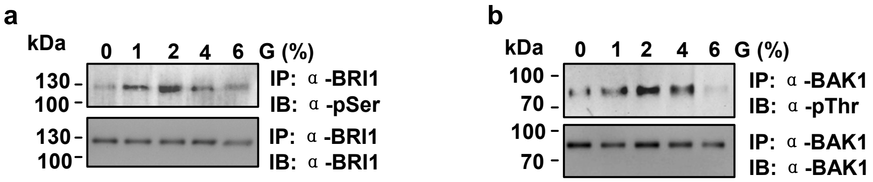

Supplementary Figure 5 | Glucose influences the phosphorylation levels of BRI1 and BAK1 in a concentration-dependent manner.

(a) Glucose influences the phosphorylation levels of BRI1 in *Arabidopsis*. Wild-type seedlings were grown under light condition for 5 days, then incubated in darkness for 4 days, and treated with glucose as indicated for 6 hours. Total proteins were isolated and incubated with anti-BRI1 antibody for 30min, and then incubated with Protein G Magnetic Beads for 30min to immunoprecipitate BRI1. Immunoprecipitated proteins were detected with anti-BRI1 and anti-phosphoserine (pSer) antibodies, respectively. IP, immunoprecipitation; IB, immunoblot; G, glucose.

(b) Glucose influences the phosphorylation levels of BAK1 in *Arabidopsis*. Wild-type seedlings were grown under light condition for 5 days, then incubated in darkness for 4 days, and treated with glucose as indicated for 6 hours. Total proteins were isolated and incubated with anti-BAK1 antibody for 30min, and then incubated with Protein G Magnetic Beads for 30min to immunoprecipitate BAK1. Immunoprecipitated proteins were detected with anti-BAK1 and anti-phosphoserine (pThr) antibodies, respectively. IP, immunoprecipitation; IB, immunoblot; G, glucose.

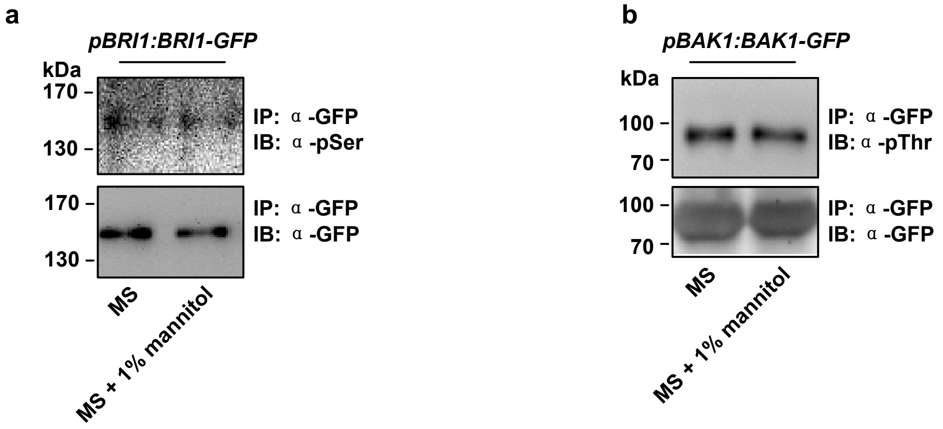

Supplementary Figure 6 | Mannitol does not affect the phosphorylation levels of BRI1 and BAK1.
(a) *pBRI1:BRI1-GFP* seedlings were grown under light condition for 5 days, then incubated in darkness for 4 days, and finally treated with 1% mannitol for 4 hours. Total proteins were isolated and incubated with green fluorescent protein (GFP)-Trap-A agarose beads to immunoprecipitate BRI1-GFP. Immunoprecipitated proteins were detected with anti-GFP and anti-phosphoserine (pSer) antibodies, respectively. IP, immunoprecipitation; IB, immunoblot.
(b) *pBAK1:BAK1-GFP* seedlings were grown under light condition for 5 days, then incubated in darkness for 4 days, and finally treated with 1% mannitol for 4 hours. Total proteins were isolated and incubated with green fluorescent protein (GFP)-Trap-A agarose beads to immunoprecipitate BAK1-GFP. Immunoprecipitated proteins were detected with anti-GFP and anti-phosphothreonine (pThr) antibodies, respectively. IP, immunoprecipitation; IB, immunoblot.

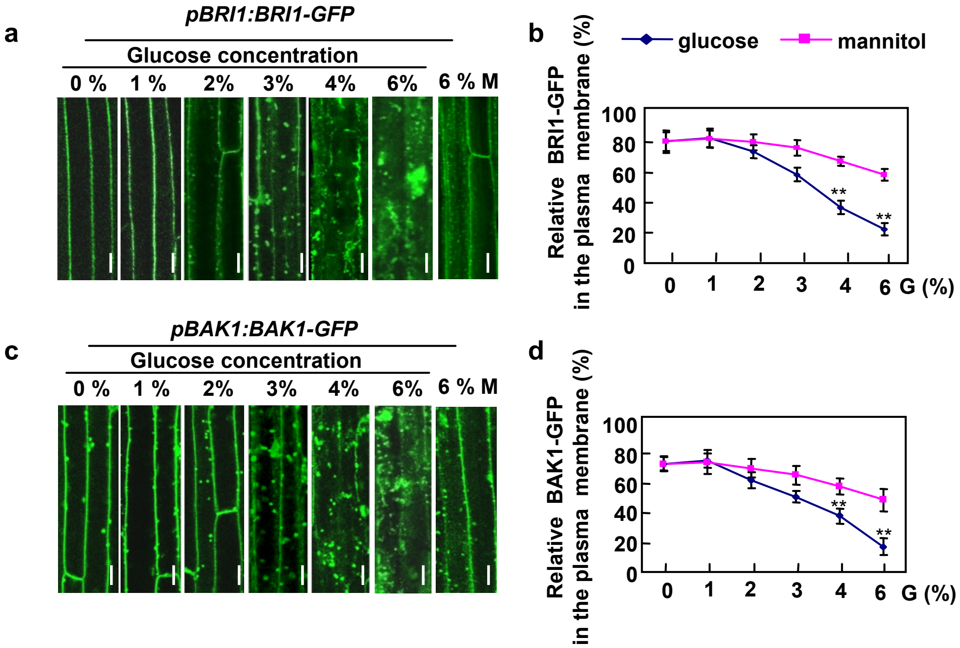

Supplementary Figure 7 | Glucose influences the plasma membrane localization of BRI1 and BAK1 in a concentration-dependent manner.

(a,b) Glucose influences the plasma membrane localization of BRI1 in *Arabidopsis*. *pBRI1:BRI1-GFP* seedlings were grown MS medium in the dark for 4 days, then treated with various concentrations of glucose (G) or mannitol (M) for 1 hour, respectively, and observed by confocal microscopy (a). Quantification of the plasma membrane localization of BRI1-GFP in hypocotyl epidermal cells treated with the indicated glucose (G) or mannitol (M) for 1 hours (b). (c,d) Glucose influences the plasma membrane localization of BAK1 in *Arabidopsis*. *pBAK1:BAK1-GFP* seedlings were grown MS medium in the dark for 4 days, then treated with various concentrations of glucose (G) or mannitol (M) for 2 hours, respectively, and observed by confocal microscopy (c). Quantification of the plasma membrane localization of BAK1-GFP in hypocotyl epidermal cells treated with the indicated glucose (G) or mannitol (M) for 2 hours (d). Values (b,d) are given as mean ± standard deviation (SD). $^{**}P < 0.01$ compared with the mannitol treatment by Student's *t*-test. Scale bars, 20 μm (a,c).

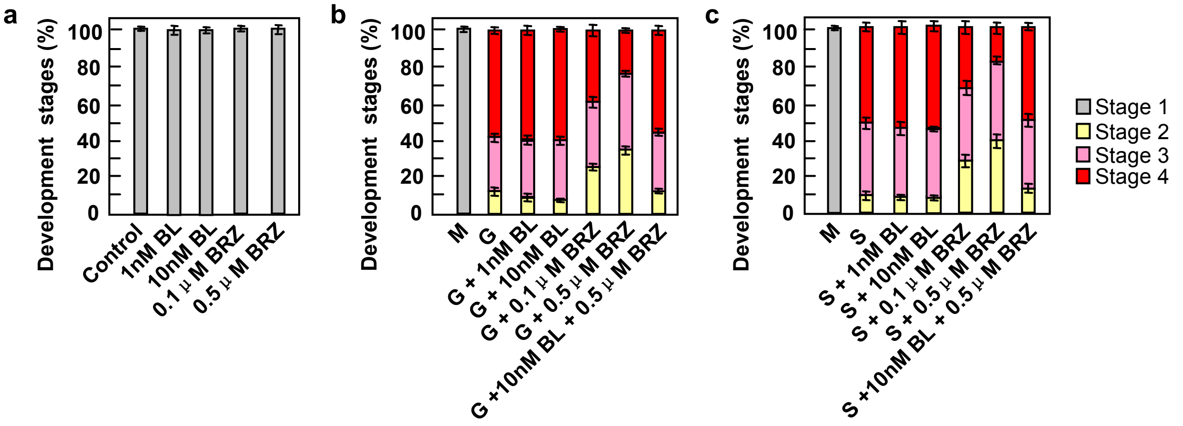

Supplementary Figure 8 | The combined effects of sugar, BL and BRZ.

(a) Comparison of developmental stages of wild type seedlings grown on MS medium supplemented with various concentrations of BRZ or BL as indicated in the dark for 19 days (n ⩾ 60). Control, MS medium without BL and BRZ.

(b) Comparison of developmental stages of wild type seedlings grown on 1% mannitol (M) medium or 1% glucose (G) medium supplemented with various concentrations of BRZ or BL as indicated in the dark for 19 days (n ⩾ 72).

(c) Comparison of developmental stages of wild type seedlings grown on 1% mannitol (M) medium or 1% sucrose (S) medium supplemented with various concentrations of BRZ or BL as indicated in the dark for 19 days (n ⩾ 69).

Values (a-c) are given as mean ± standard deviation (SD).

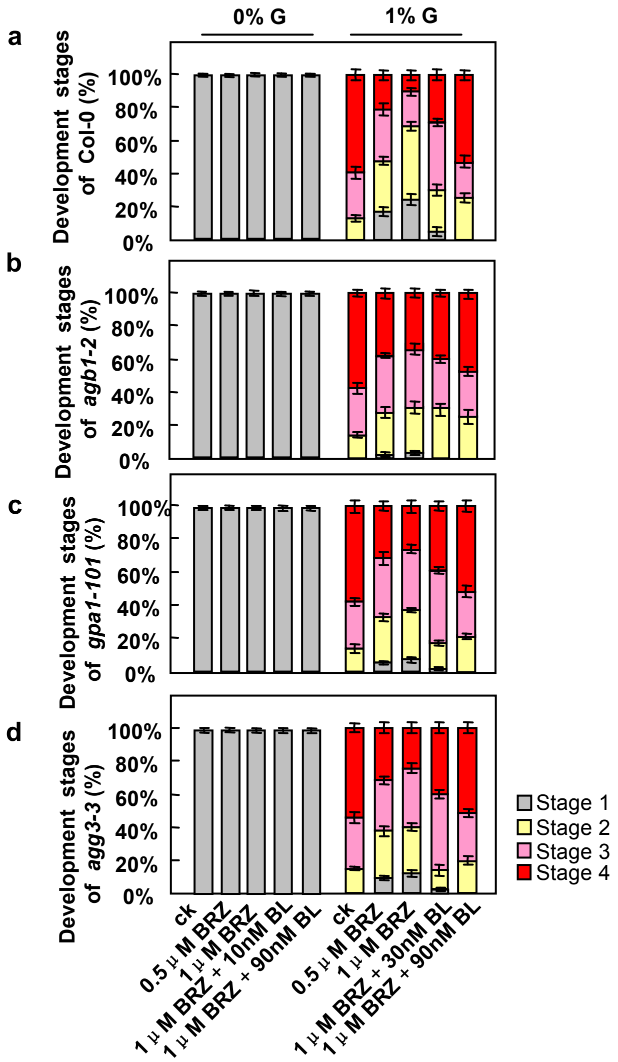

Supplementary Figure 9 | The effect of G-proteins on the crosstalk between BR and sugar in the dark-development.

(a-d) Comparison of development stages of Col-0 (a), *agb1-2* (b), *gpa1-101* (c), and *agg3-3* (d) seedlings grown on 0% or 1% glucose (G) with various concentrations of BRZ and BL as indicated in the dark for 19 days (n ⩾ 62).

Values (a-d) are given as mean ± standard deviation (SD).

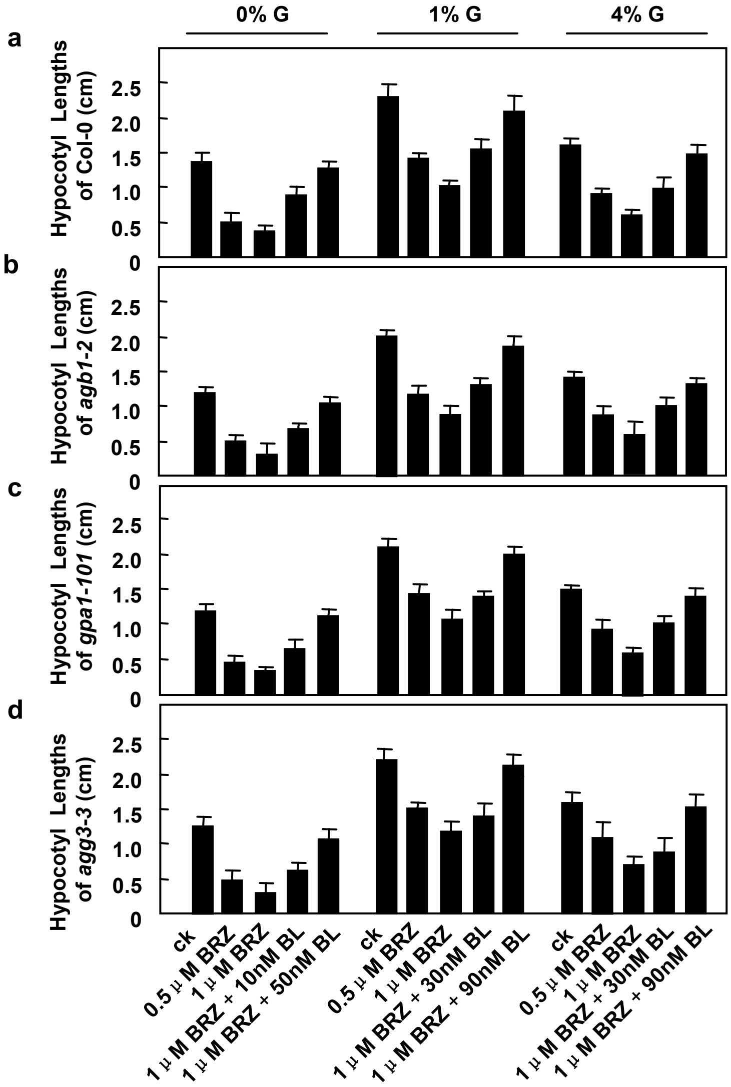

Supplementary Figure 10 | The effect of G-proteins on the crosstalk between BR and sugar in the hypocotyl elongation.

(a-d) Hypocotyl lengths of Col-0 (a), *agb1-2* (b), *gpa1-101* (c), and *agg3-3* (d) seedlings grown on 0%,1% or 4% glucose (G) medium with various concentrations of BRZ and BL as indicated in the dark for 11 days (n ⩾ 70).

Values (a-d) are given as mean ± standard deviation (SD).

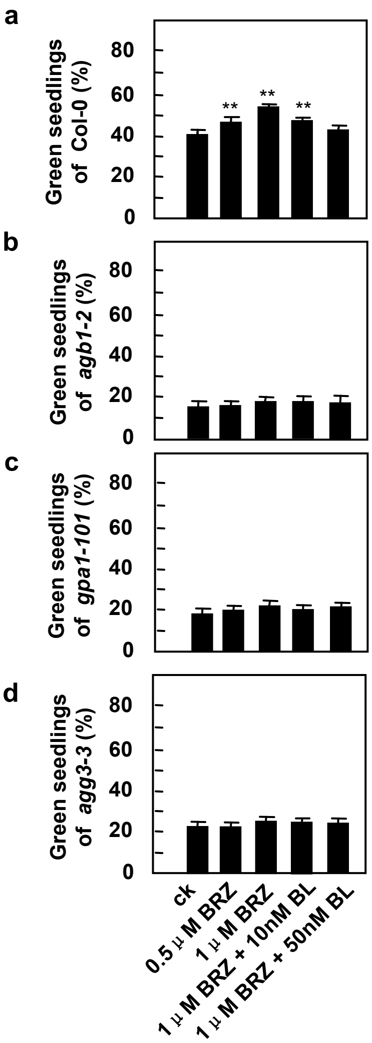

Supplementary Figure 11 | The effect of G-proteins on the crosstalk between BR and sugar in high glucose responses.

(a-d) The percentage of Col-0 (a), *agb1-2* (b), *gpa1-101* (c), and *agg3-3* (d) with green cotyledons. Seedlings were grown on 6% glucose with various concentrations of BRZ and BL as indicated under constant light condition for 15 days (n ⩾ 59).

Values (a-d) are given as mean ± standard deviation (SD).

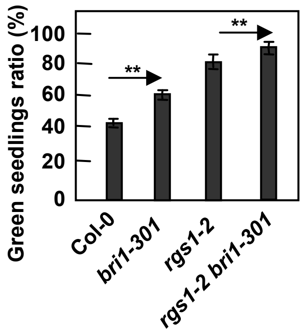

Supplementary Figure 12 | BRI1 acts redundantly with RGS1 to control sugar responses.
The percentages of Col-0,*bri1-301*,*rgs1-2* and *bri1-301 rgs1-2* seedlings with green cotyledons. Seedlings were grown on MS medium with 6% glucose under constant light condition for 16 days (n ⩾ 71).
Values are given as mean ± standard deviation (SD). ***P* < 0.01 compared with the respective control by Student's *t*-test.

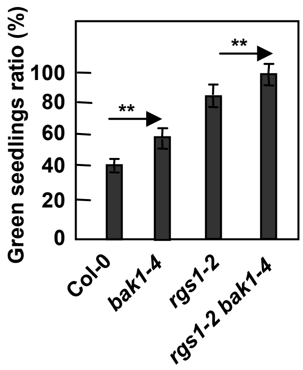

Supplementary Figure 13 | BAK1 acts redundantly with RGS1 to control sugar responses.
The percentages of Col-0*, bak1-4*, *rgs1-2* and *bak1-4 rgs1-2* seedlings with green cotyledons. Seedlings were grown on MS medium with 6% glucose under constant light condition for 16 days (n ⩾ 63).
Values are given as mean ± standard deviation (SD). $^{**}P$ < 0.01 compared with the respective control by Student's *t*-test.

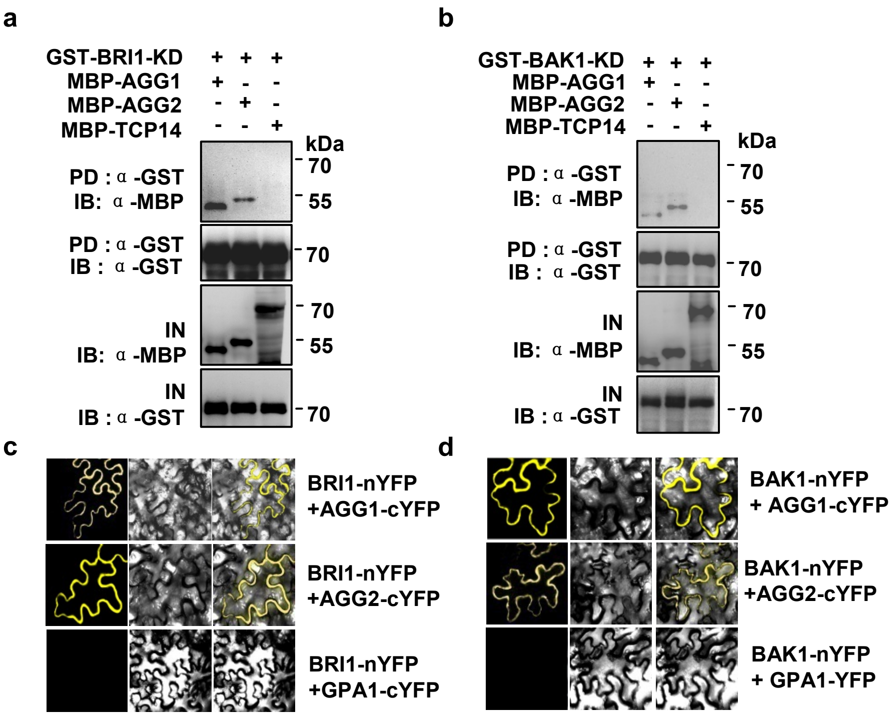

Supplementary Figure 14 | BRI1 and BAK1 interact with AGG1 and AGG2, respectively.

(a) BRI1 kinase domain (BRI1-KD) physically interacts with AGG1 and AGG2 *in vitro*. MBP-TCP14, MBP-AGG1 or MBP-AGG2 was pulled down (PD) by GST-BRI1-KD immobilized on GST beads and analyzed by immunoblotting (IB) using an anti-MBP antibody. IN, input. MBP-TCP14 is a negative control.

(b) BAK1 kinase domain (BAK1-KD) physically interacts with AGG1 and AGG2 *in vitro*. MBP-TCP14, MBP-AGG1 or MBP-AGG2 was pulled down (PD) by GST-BAK1-KD immobilized on GST beads and analyzed by immunoblotting (IB) using an anti-MBP antibody. IN, input. MBP-TCP14 is a negative control.

(c) Bimolecular fluorescence complementation (BiFC) assays show that BRI1 interacts with AGG1 and AGG2 in *N.benthamiana* leaves. BRI1-nYFP was coexpressed with AGG1-cYFP, AGG2-cYFP or GPA1-cYFP in leaves of *N. benthamiana.*

(d) Bimolecular fluorescence complementation (BiFC) assays show that BAK1 interacts with AGG1 and AGG2 in *N.benthamiana* leaves. BAK1-nYFP was coexpressed with AGG1-cYFP, AGG2-cYFP or GPA1-cYFP in leaves of *N. benthamiana*.

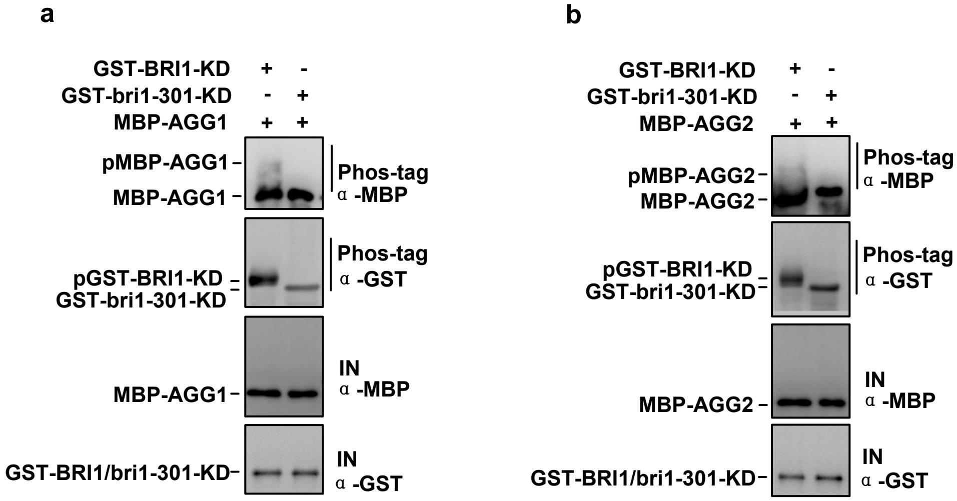

Supplementary Figure 15 | BRI1 phosphorylates AGG1 and AGG2.
(a) BRI1 kinase domain (BRI1-KD) phosphorylates AGG1 in *vitro,* but bri1-301 kinase domain (bri1-301-KD) does not. MBP-AGG1 was incubated with GST-BRI1-KD and GST-bri1-301-KD in an *in vitro* kinase assay buffer. The phosphorylated MBP-AGG1 (pMBP-AGG1) and unphosphorylated (MBP-AGG1) were separated by 10% SDS–PAGE with phos-tag, and then immunobloted with anti-MBP antibody. IN, input.
(b) BRI1 kinase domain (BRI1-KD) phosphorylates AGG2 *in vitro,* but bri1-301 kinase domain (bri1-301-KD) does not. MBP-AGG2 was incubated with GST-BRI1-KD and GST-bri1-301-KD in an *in vitro* kinase assay buffer. The phosphorylated MBP-AGG2 (pMBP-AGG2) and unphosphorylated (MBP-AGG2) were separated by 10% SDS–PAGE with phos-tag, and then immunobloted with anti-MBP antibody. IN, input.

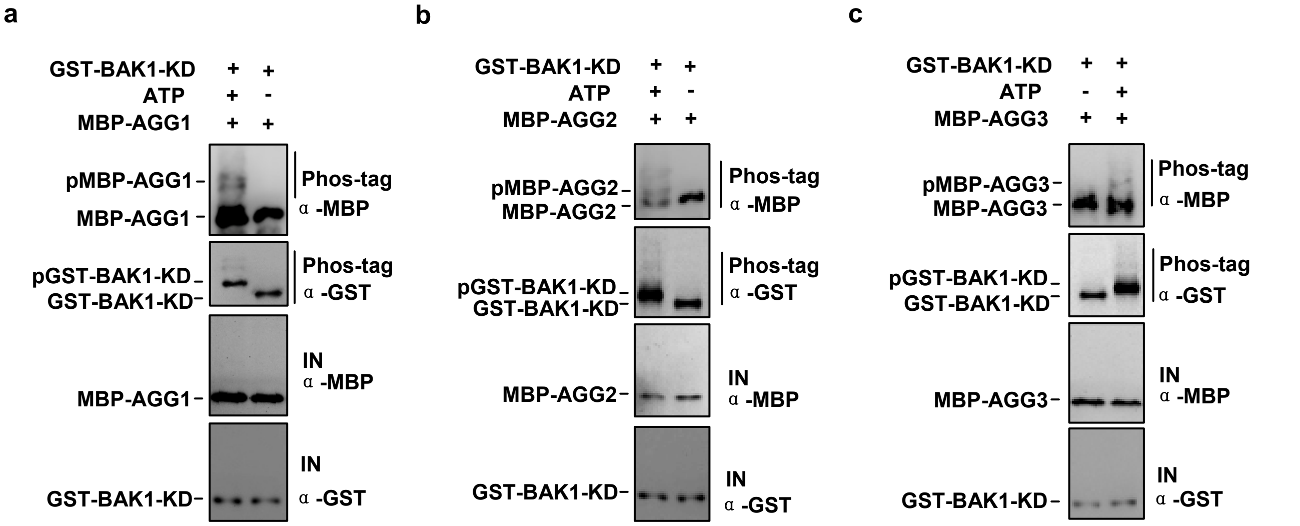

Supplementary Figure 16｜BAK1 phosphorylates AGG1, AGG2 and AGG3.

(a) BAK1 kinase domain (BAK1-KD) phosphorylates AGG1 in the presence of ATP, but does not in the absence of ATP. MBP-AGG1 was incubated with GST-BAK1-KD in an *in vitro* kinase assay buffer. The phosphorylated MBP-AGG1 (pMBP-AGG1) and unphosphorylated (MBP-AGG1) were separated by 10% SDS–PAGE with phos-tag, and then immunobloted with anti-MBP antibody. IN, input.

(b) BAK1 kinase domain (BAK1-KD) phosphorylates AGG2 in the presence of ATP, but does not in the absence of ATP. MBP-AGG1 was incubated with GST-BAK1-KD in an *in vitro* kinase assay buffer. The phosphorylated MBP-AGG2 (pMBP-AGG2) and unphosphorylated (MBP-AGG2) were separated by 10% SDS–PAGE with phos-tag, and then immunobloted with anti-MBP antibody. IN, input.

(c) BAK1 kinase domain (BAK1-KD) phosphorylates AGG3 in the presence of ATP, but does not in the absence of ATP. MBP-AGG3 was incubated with GST-BAK1-KD in an *in vitro* kinase assay buffer. The phosphorylated MBP-AGG3 (pMBP-AGG3) and unphosphorylated (MBP-AGG3) were separated by 10% SDS–PAGE with phos-tag, and then immunobloted with anti-MBP antibody. IN, input.

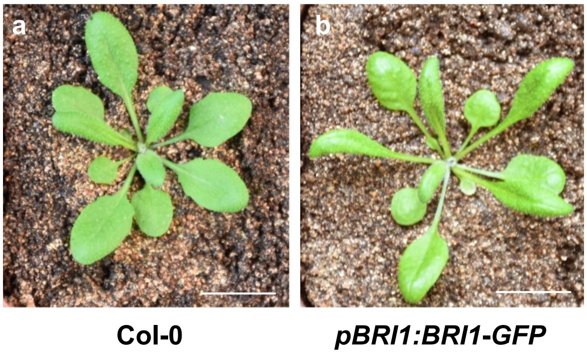

Supplementary Figure 17 | *pBRI1:BRI1-GFP* plants show the phenotypes of *BRI1*-overexpressing lines.
(a) 21-d-old Col-0 plants. (Scale bar: 2 cm.)
(b) 21-d-old *proBRI:BRI1-GFP* plants. *pBRI:BRI1-GFP* plants exhibited long petioles and dome-shaped leaves, like those observed in plants overexpressing *BRI1*, indicating that *pBRI:BRI1-GFP* plants have increased activity of BRI1. (Scale bar, 2 cm.)

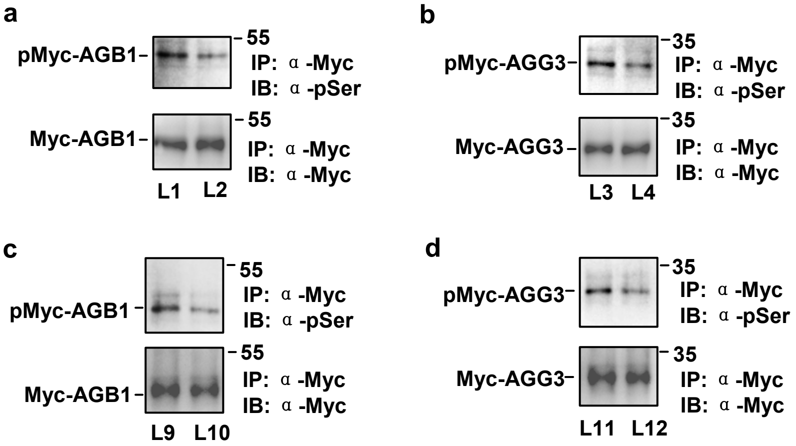

Supplementary Figure 18 | BRI1 influences the phosphorylation of AGB1 and AGG3 in *Arabidopsis.*
(a) BRI1 influences the phosphorylation of AGB1 in *Arabidopsis*. Total proteins from *pBRI1:BRI1-GFP*;*35S:Myc-AGB1* (L1) and *35S:GFP*;*35S:Myc-AGB1*(L2) seedlings were immunoprecipitated (IP) with anti-Myc-Tag mouse mAb conjugated agarose beads, and the immunoblot (IB) was probed with anti-Myc and anti-phosphoserine (pSer) antibodies, respectively. pMyc-AGB1, phosphorylated Myc-AGB1; IP, immunoprecipitation; IB, immunoblot.
(b) BRI1 influences the phosphorylation of AGG3 in *Arabidopsis*. Total proteins from *pBRI1:BRI1-GFP*;*35S:Myc-AGG3* (L3) and *35S:GFP*;*35S:Myc-AGG3* (L4) seedlings were immunoprecipitated (IP) with anti-Myc-Tag mouse mAb conjugated agarose beads, and the immunoblot (IB) was probed with anti-Myc and anti-phosphoserine (pSer) antibodies, respectively. pMyc-AGG3, phosphorylated Myc-AGG3; IP, immunoprecipitation; IB, immunoblot.
(c) BRI1 influences the phosphorylation of AGB1 in *Arabidopsis*. Total proteins from *35S:Myc-AGB1* (L9) and *35S:Myc-AGB1*;*bri1-301* (L10) seedlings were immunoprecipitated (IP) with anti-Myc-Tag mouse mAb conjugated agarose beads, and the immunoblot (IB) was probed with anti-Myc and anti-phosphoserine (pSer) antibodies, respectively. pMyc-AGB1, phosphorylated Myc-AGB1; IP, immunoprecipitation; IB, immunoblot.
(d) BRI1 influences the phosphorylation of AGG3 in *Arabidopsis*. Total proteins from *35S:Myc-AGG3* (L11) and *35S:Myc-AGG3*;*bri1-301* (L12) seedlings were immunoprecipitated (IP) with anti-Myc-Tag mouse mAb conjugated agarose beads, and the immunoblot (IB) was probed with anti-Myc and anti-phosphoserine (pSer) antibodies, respectively. pMyc-AGG3, phosphorylated Myc-AGG3; IP, immunoprecipitation; IB, immunoblot.

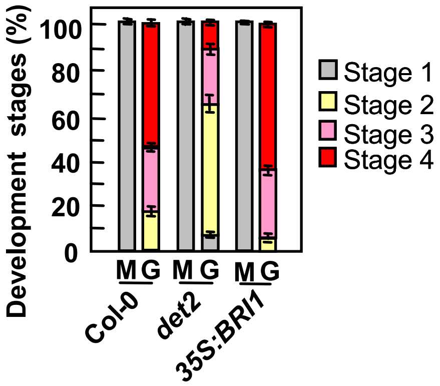

Supplementary Figure 19 | Dark development *of 35S:BRI1* and *det2* in response to glucose. Comparison of developmental stages between Col-0, *det2* and *35S:BRI1.* Seedlings were grown on MS medium with 1% glucose (G) or 1% mannitol (M) in the dark for 19 days (n ⩾ 63). Values are given as mean ± standard deviation (SD).

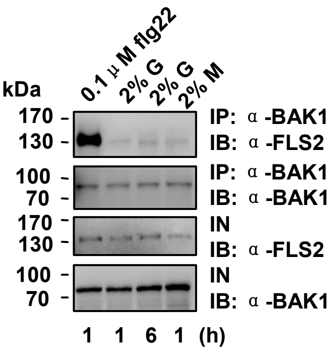

Supplementary Figure 20 | Glucose does not influence the interactions between FLS2 and BAK1 in plants.

Glucose does not influence the interactions between FLS2 and BAK1. Wild-type seedlings were grown under light condition for 5 days, then incubated in darkness for 4 days, and treated as indicated for 1 or 6 hours. Total proteins were isolated and incubated with anti-BAK1 antibody for 30min, and then incubated with Protein G Magnetic Beads for 30min to immunoprecipitate BAK1. Immunoprecipitated proteins were detected with anti-FLS2 and anti-BAK1 antibodies, respectively. IN, input; IP, immunoprecipitation; IB, immunoblot; G, glucose. M, mannitol..

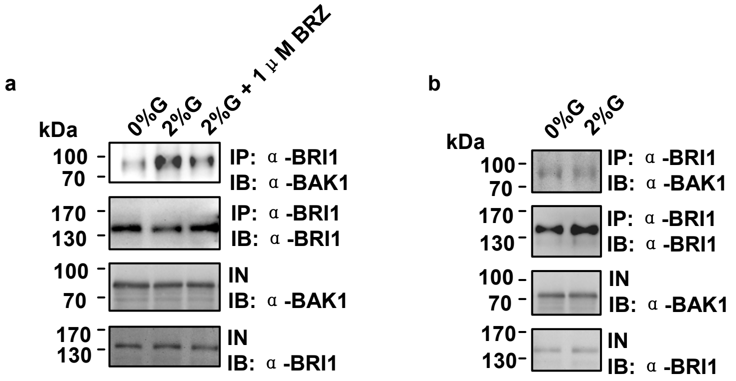

Supplementary Figure 21｜BRZ inhibits the interaction between BRI1 and BAK1 induced by glucose.

(a) BRZ inhibits the interaction between BRI1 and BAK1 induced by glucose (G). Wild-type seedlings were grown vertically on MS medium under light condition for 5 days, incubated in darkness for 4 days, and then treated with or without 2% glucose, or with 2% glucose plus 1 μM BRZ for 6 hours. Total proteins were isolated and incubated with anti-BRI1 antibody for 30min, then incubated with Protein G Magnetic Beads for 30min to immunoprecipitate BRI1. Immunoprecipitated proteins were detected with anti-BRI1 and anti-BAK1 antibodies, respectively. IN, input; IP, immunoprecipitation; IB, immunoblot.

(b) BRZ inhibits the interaction of BRI1 and BAK1 induced by glucose (G). Wild type seedlings were grown vertically on MS medium supplemented with 1 μM BRZ under light condition for 5 days, incubated in darkness for 4 days, and then treated with or without 2% glucose for 6 hours. Total proteins were isolated and incubated with anti-BRI1 antibody for 30min, then incubated with Protein G Magnetic Beads for 30min to immunoprecipitate BRI1. Immunoprecipitated proteins were detected with anti-BRI1 and anti-BAK1 antibodies, respectively. IN, input; IP, immunoprecipitation; IB, immunoblot.

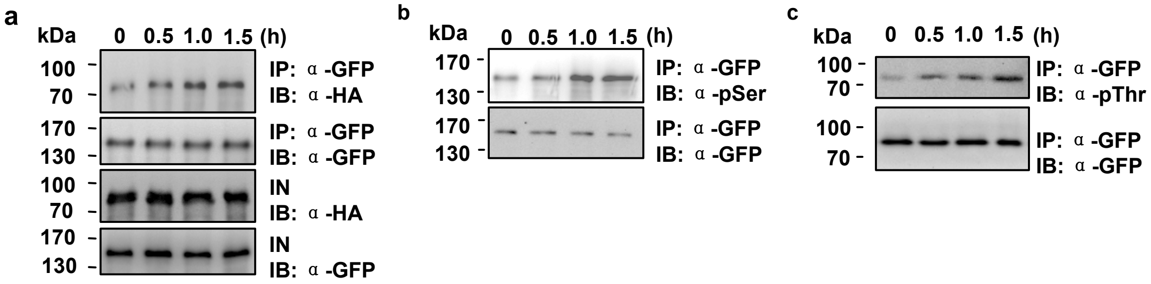

Supplementary Figure 22. Glucose influences the interactions and phosphorylations of BRI1 and BAK1 in short time.

(a) Glucose influences the interactions between BRI1 and BAK1 in short time. *pBRI1:BRI1-GFP*;*35S:BAK1-HA* seedlings were grown under light condition for 5 days, then incubated in darkness for 4 days, and treated with 2% glucose for 0, 0.5, 1.0, or 1.5 hours, respectively. Total proteins were isolated and incubated with green fluorescent protein (GFP)-Trap-A agarose beads to immunoprecipitate BRI1-GFP. Immunoprecipitated proteins were detected with anti-GFP and anti-HA antibodies, respectively. IN, input; IP, immunoprecipitation; IB, immunoblot.

(b) Glucose influences the phosphorylation levels of BRI1 in short time. *pBRI1:BRI1-GFP* seedlings were grown under light condition for 5 days, then incubated in darkness for 4 days, and treated with 2% glucose for 0, 0.5, 1.0, or 1.5 hours, respectively. Total proteins were isolated and incubated with green fluorescent protein (GFP)-Trap-A agarose beads to immunoprecipitate BRI1-GFP. Immunoprecipitated proteins were detected with anti-GFP and anti-phosphoserine (pSer) antibodies, respectively. IN, input; IP, immunoprecipitation; IB, immunoblot.

(c) Glucose influences the phosphorylation levels of BAK1 in short time. *pBAK1:BAK1-GFP* seedlings were grown under light condition for 5 days, then incubated in darkness for 4 days, and treated with 2% glucose for 0, 0.5, 1.0, or 1.5 hours, respectively. Total proteins were isolated and incubated with green fluorescent protein (GFP)-Trap-A agarose beads to immunoprecipitate BAK1-GFP. Immunoprecipitated proteins were detected with anti-GFP and anti-phosphothreonine (pThr) antibodies, respectively. IN, input; IP, immunoprecipitation; IB, immunoblot.

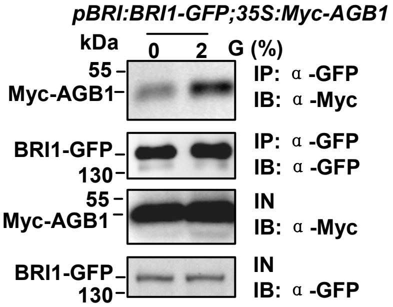

Supplementary Figure 23 | Effects of glucose on the interactions between BRI1 and AGB1 in *Arabidopsis*.

Glucose regulates the interactions between BRI1 and AGB1 in *Arabidopsis*. *pBRI1:BRI1-GFP*;*35S:Myc-AGB1* seedlings were grown under light condition for 5 days, then incubated in darkness for 4 days, and treated without or with 2% glucose (G) for 4 hours. Total proteins were isolated and incubated with green fluorescent protein (GFP)-Trap-A agarose beads to immunoprecipitate BRI1-GFP. Immunoprecipitated proteins were detected with anti-GFP and anti-Myc antibodies, respectively. IN, input; IP, immunoprecipitation; IB, immunoblot.

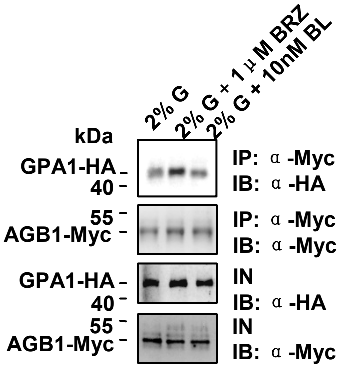

Supplementary Figure 24. Effects of BL and BRZ on the glucose-induced interactions between GPA1 and AGB1.
Arabidopsis leaf protoplasts of the wild type were co-transformed by injection of *Agrobacterium* GV3101 cells harboring *35S:AGB1-Myc* and *35S*:*GPA1-HA* plasmids, grown in darkness for 14 hours, and then treated as indicated for 5 hours. Total proteins from leaf protoplasts were immunoprecipitated with anti-Myc-Tag mouse mAb conjugated agarose beads, and the immunoblot (IB) was probed with anti-Myc and anti-HA antibodies, respectively. IN, input; IP, immunoprecipitation; G, glucose.

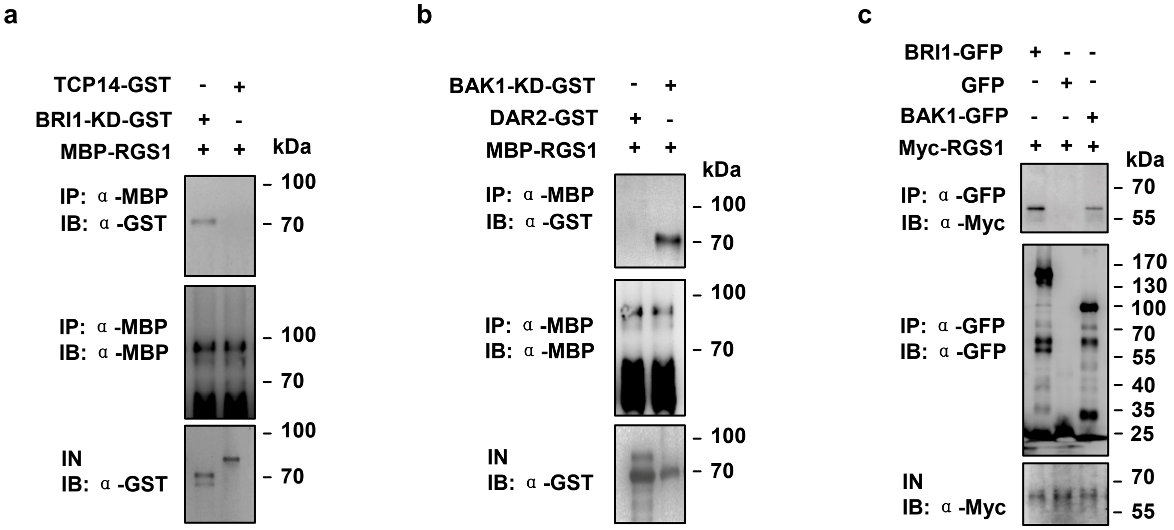

Supplementary Figure 25 | BRI1 and BAK1 physically interact with RGS1.

(a) BRI1 kinase domain (BRI1-KD) physically interacts with RGS1 *in vitro*. MBP-RGS1 was pulled down (PD) by GST-BRI1-KD immobilized on GST beads and analyzed by immunoblotting (IB) using an anti-MBP antibody. IN, input.GST-TCP14 is a negative control.

(b) BAK1 kinase domain (BAK1-KD) physically interacts with RGS1 *in vitro*. MBP-RGS1was pulled down (PD) by GST-BAK1-KD immobilized on GST beads and analyzed by immunoblotting (IB) using an anti-MBP antibody. IN, input. GST-DAR2 is a negative control.

(c) BRI1 and BAK1 interacts with RGS1 in vivo. *pBRI1:BRI1-GFP*; *35S:Myc-RGS1* or *pBAK:BAK-GFP*; *35S:Myc-RGS1* seedlings were used in this assay. Total proteins were immunoprecipitated with GFP-Trap-A, and the immunoblots were probed with anti-GFP and anti-Myc antibodies. Myc-RGS1 were detected in the immunoprecipitated BRI1-GFP or BAK1-GFP complexrespectively. IN, input; IP, immunoprecipitation; IB, immunoblot.

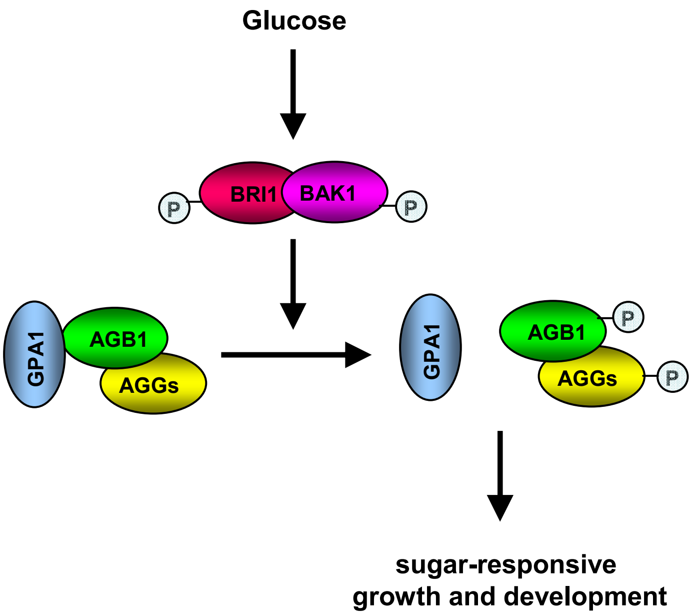

Supplementary Figure 26 | A model of sugar signal pathway mediated by BRI1/ BAK1
Sugar and BR crosstalks act through G-proteins to regulate sugar-responsive growth and development. Low concentrations of glucose promote the physical interactions between BRI1 and BAK1 and the phosphorylation levels of BRI1 and BAK1. The activated BRI1/ BAK1 complex phosphorylates AGB1 and AGGs, resulting in the dissociation of Gα and Gβ/γ and the activation of G protein signaling. The activated G protein signaling modulates sugar-responsive growth and development.

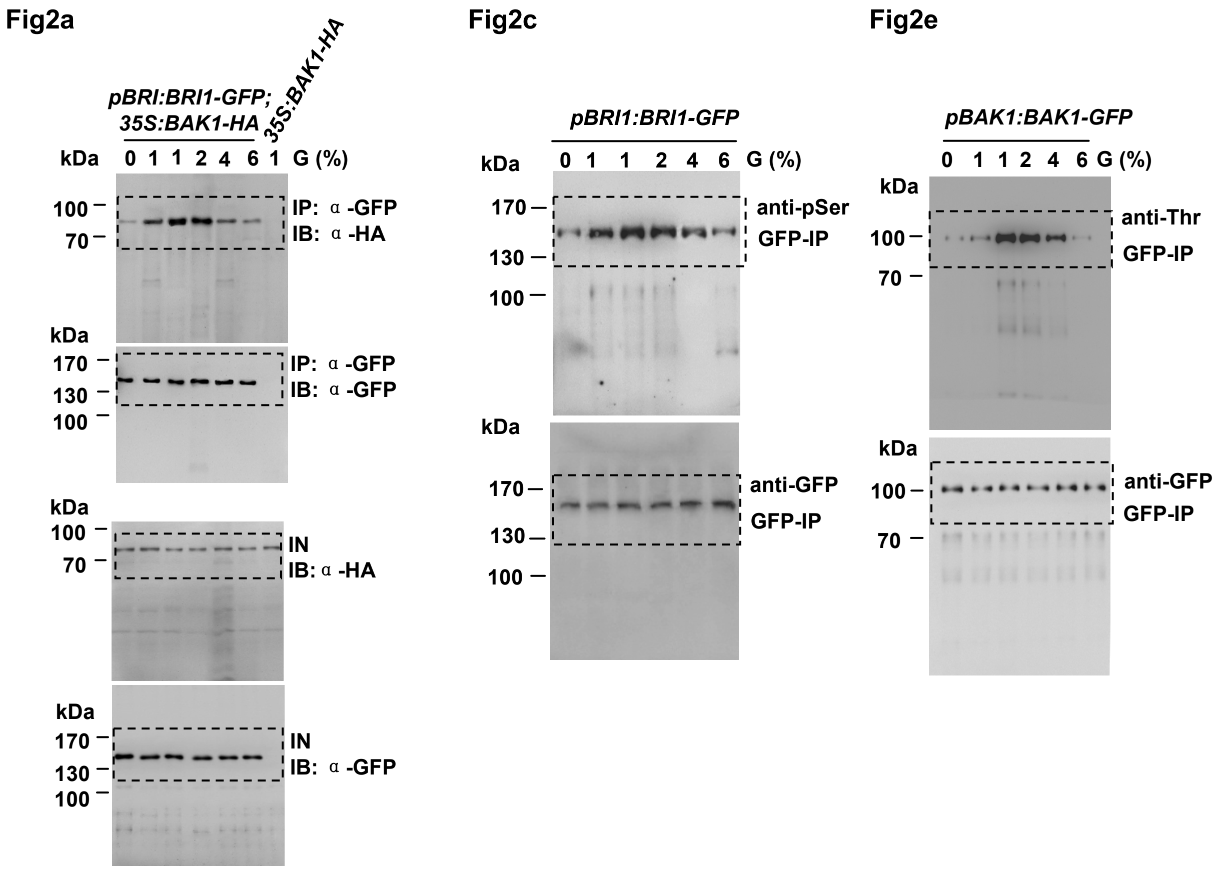

Supplementary Figure27 | Uncropped images of blots shown in Fig. 2.

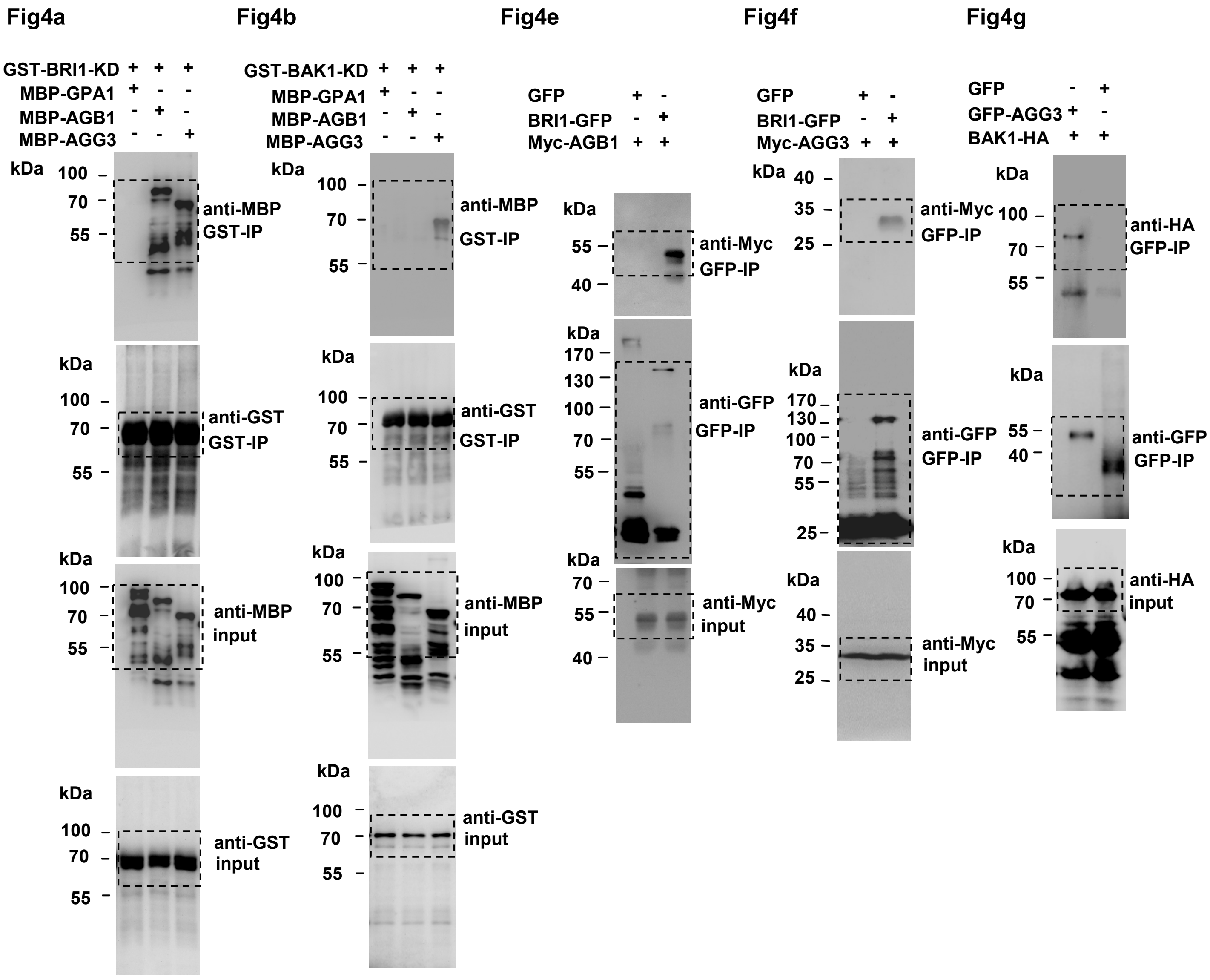

Supplementary Figure28 | Uncropped images of blots shown in Fig. 4.

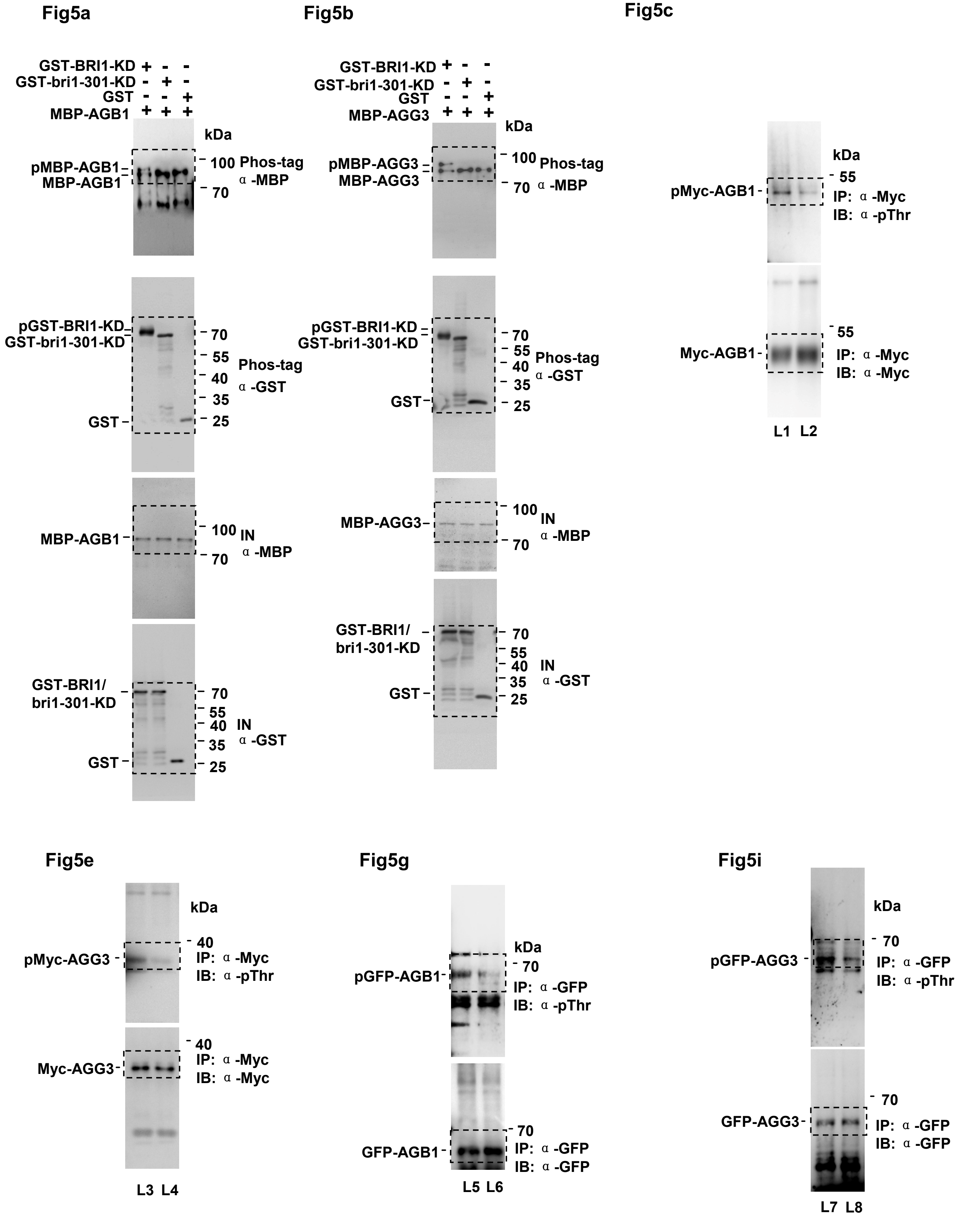

Supplementary Figure29 | Uncropped images of blots shown in Fig. 5.

Fig7a

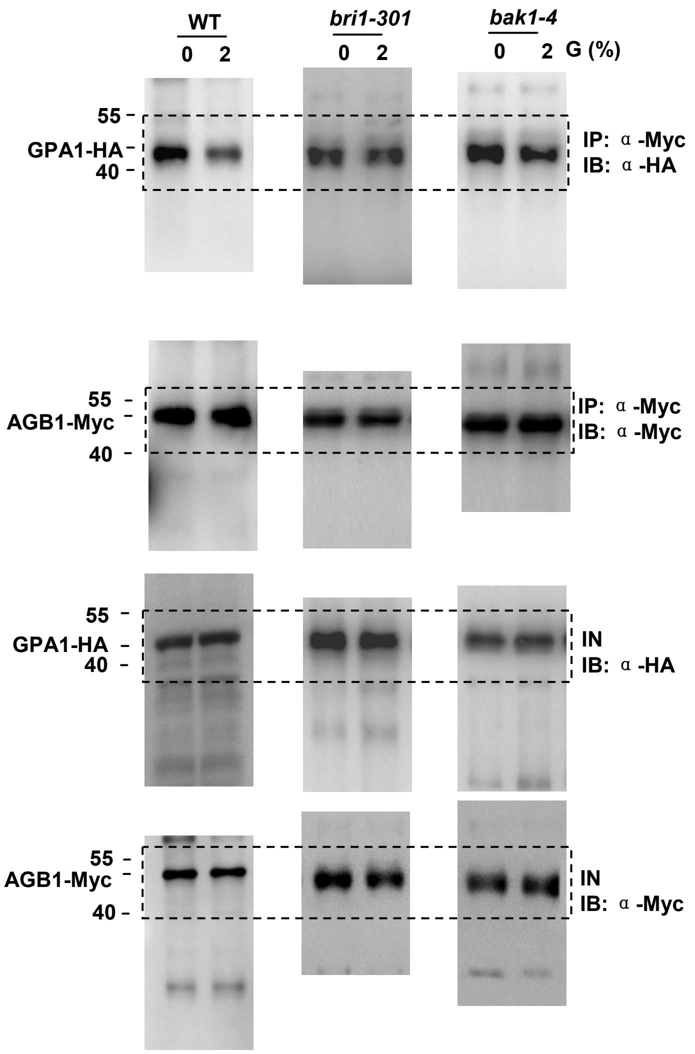

Supplementary Figure30 | Uncropped images of blots shown in Fig. 7.

Supplementary Fig 3

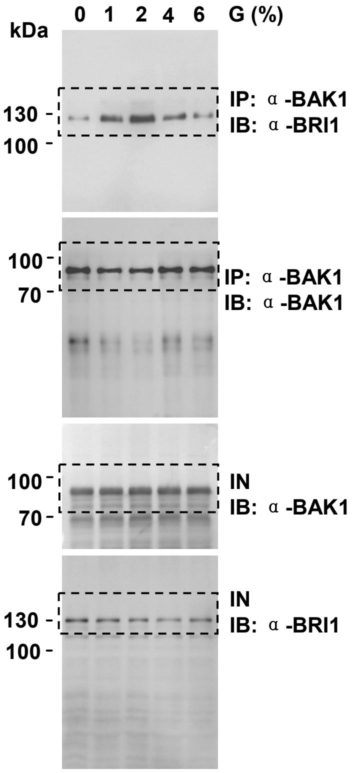

Supplementary Fig 4

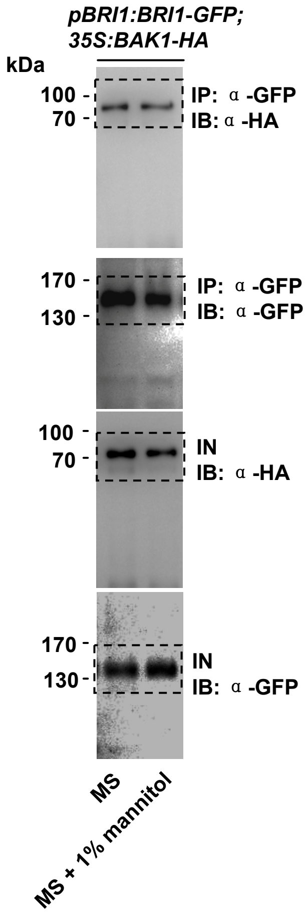

Supplementary Fig 5a

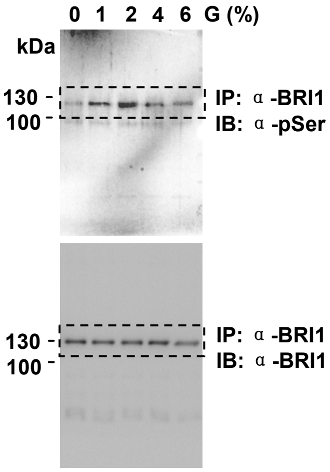

Supplementary Fig 5b

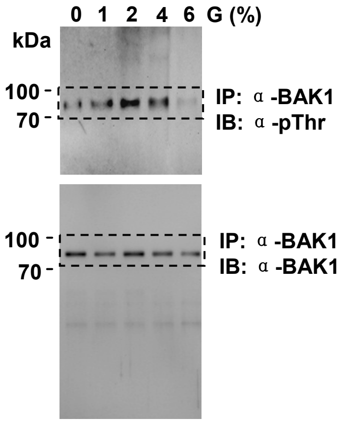

Supplementary Fig 6a

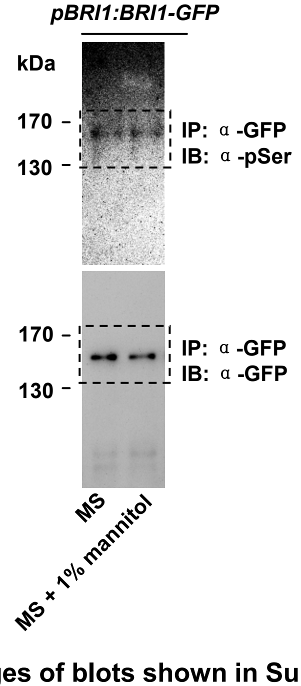

Supplementary Fig 6b

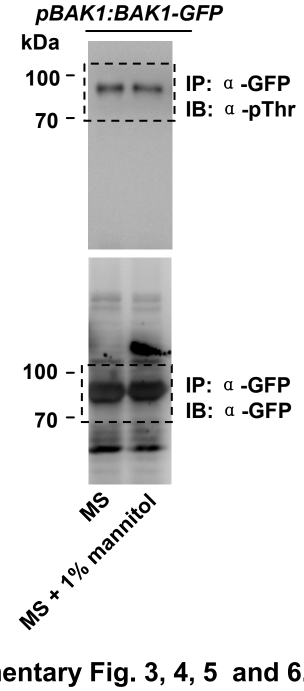

Supplementary Figure31 | Uncropped images of blots shown in Supplementary Fig. 3, 4, 5 and 6.

Supplementary Fig 14a

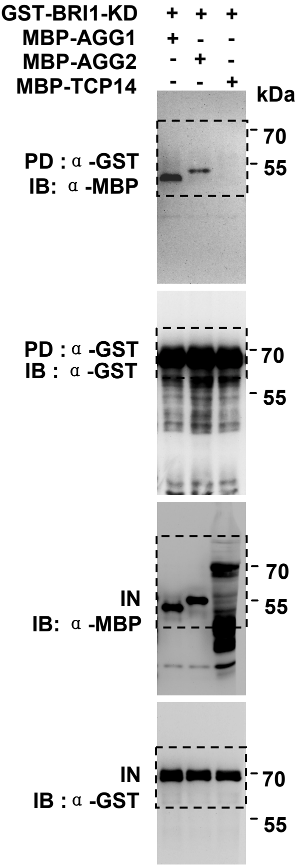

Supplementary Fig 14b

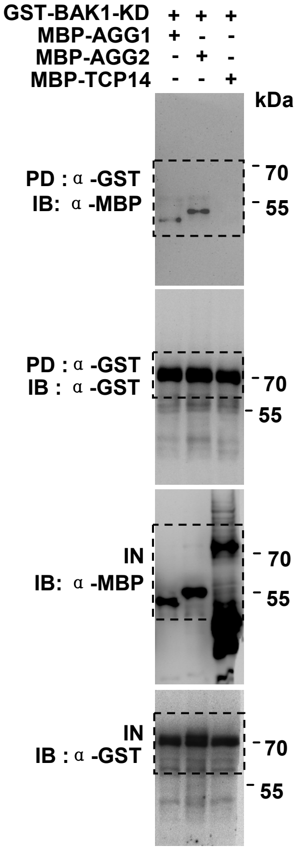

Supplementary Fig 15a

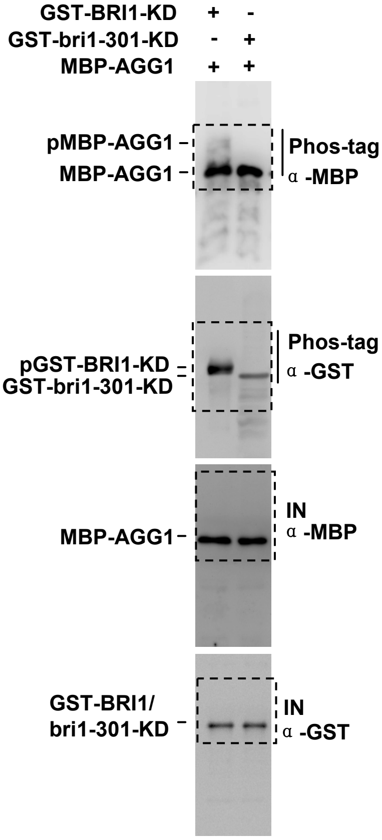

Supplementary Fig 15b

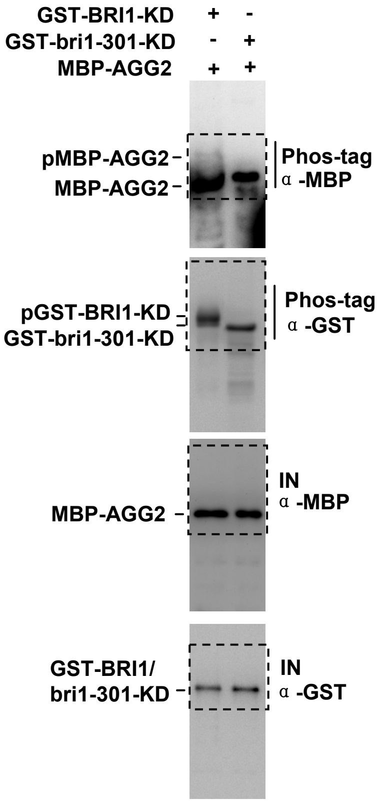

Supplementary Fig 16a

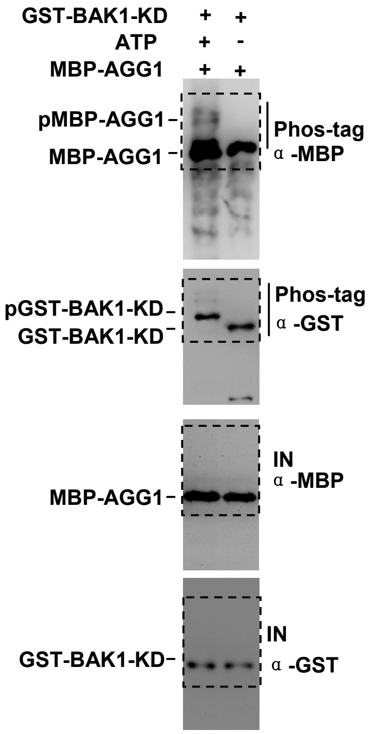

Supplementary Fig 16b

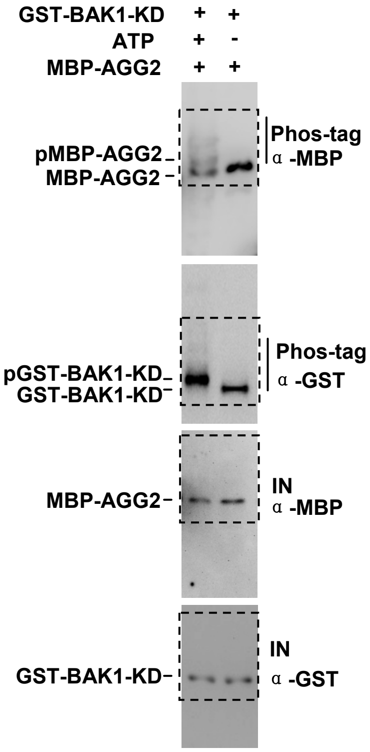

Supplementary Fig 16c

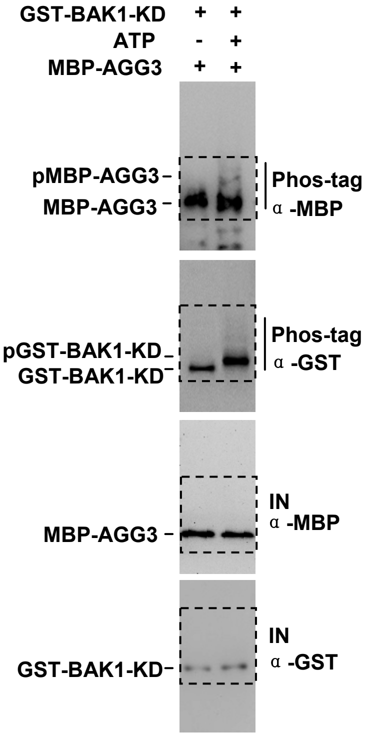

Supplementary Figure32 | Uncropped images of blots shown in Supplementary Fig. 14, 15 and 16.

Supplementary Fig 18a

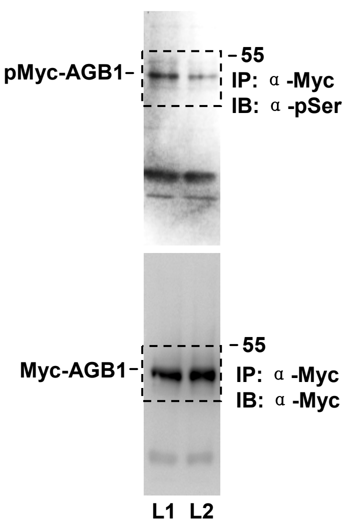

Supplementary Fig 18b

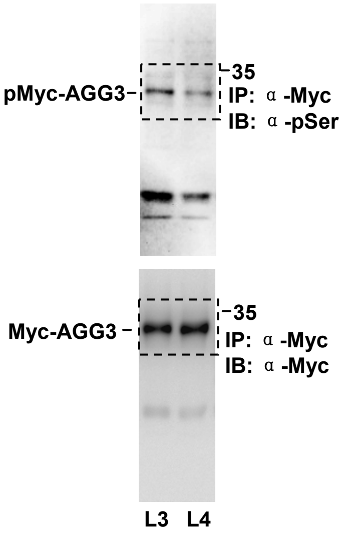

Supplementary Fig 18c

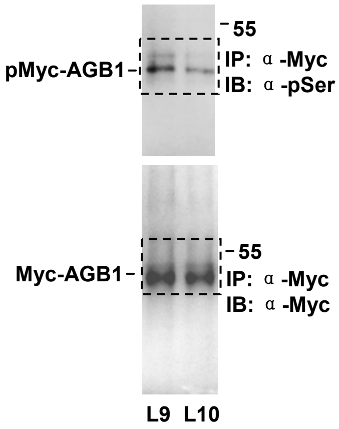

Supplementary Fig 18d

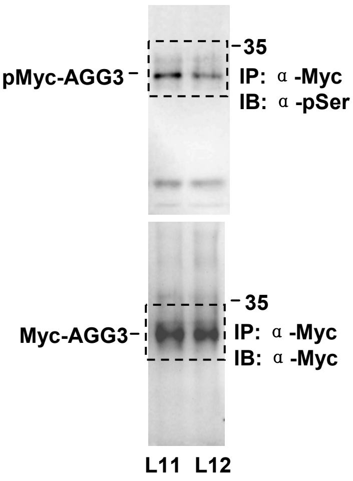

Supplementary Fig 20

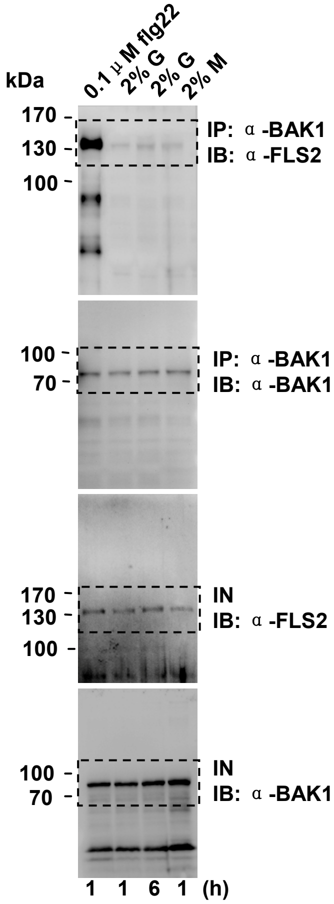

Supplementary Fig 21a

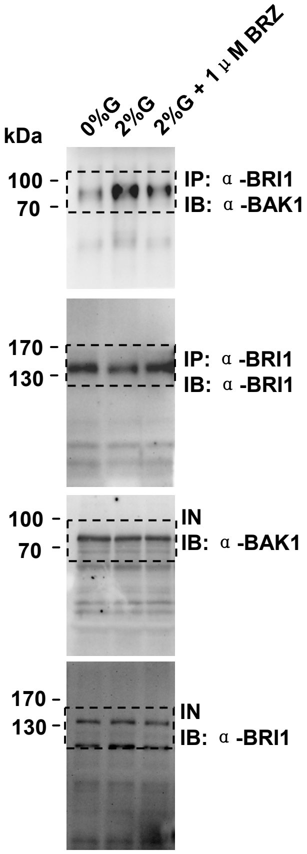

Supplementary Fig 21b

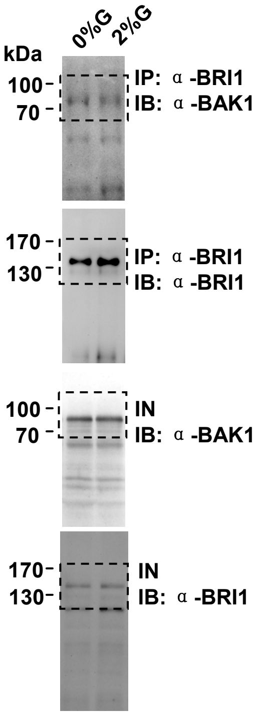

Supplementary Figure33 | Uncropped images of blots shown in Supplementary Fig. 18, 20 and 21.

Supplementary Fig 22a

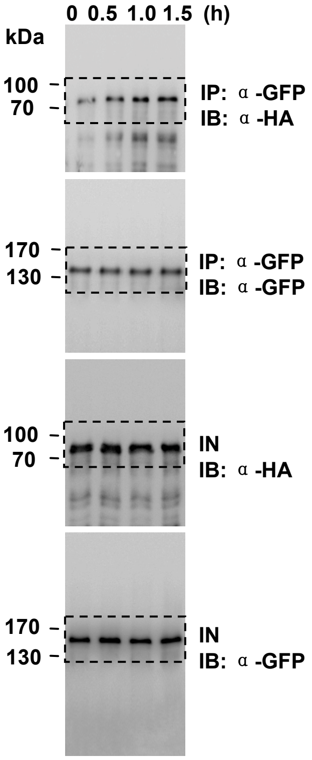

Supplementary Fig 22b

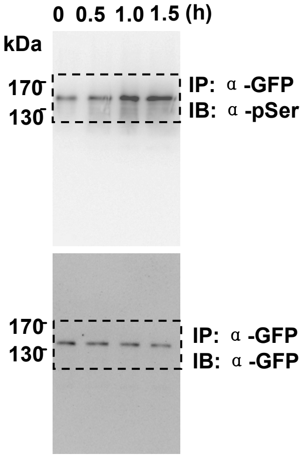

Supplementary Fig22c

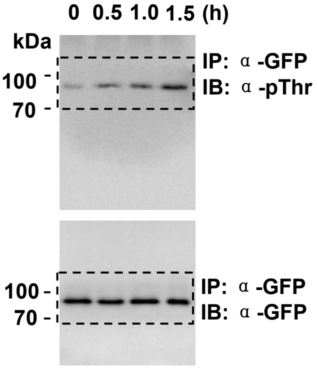

Supplementary Fig 23

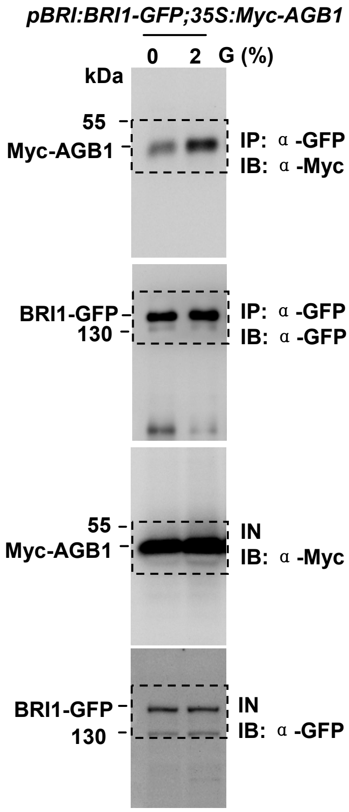

Supplementary Figure34 | Uncropped images of blots shown in Supplementary Fig. 22 and 23.

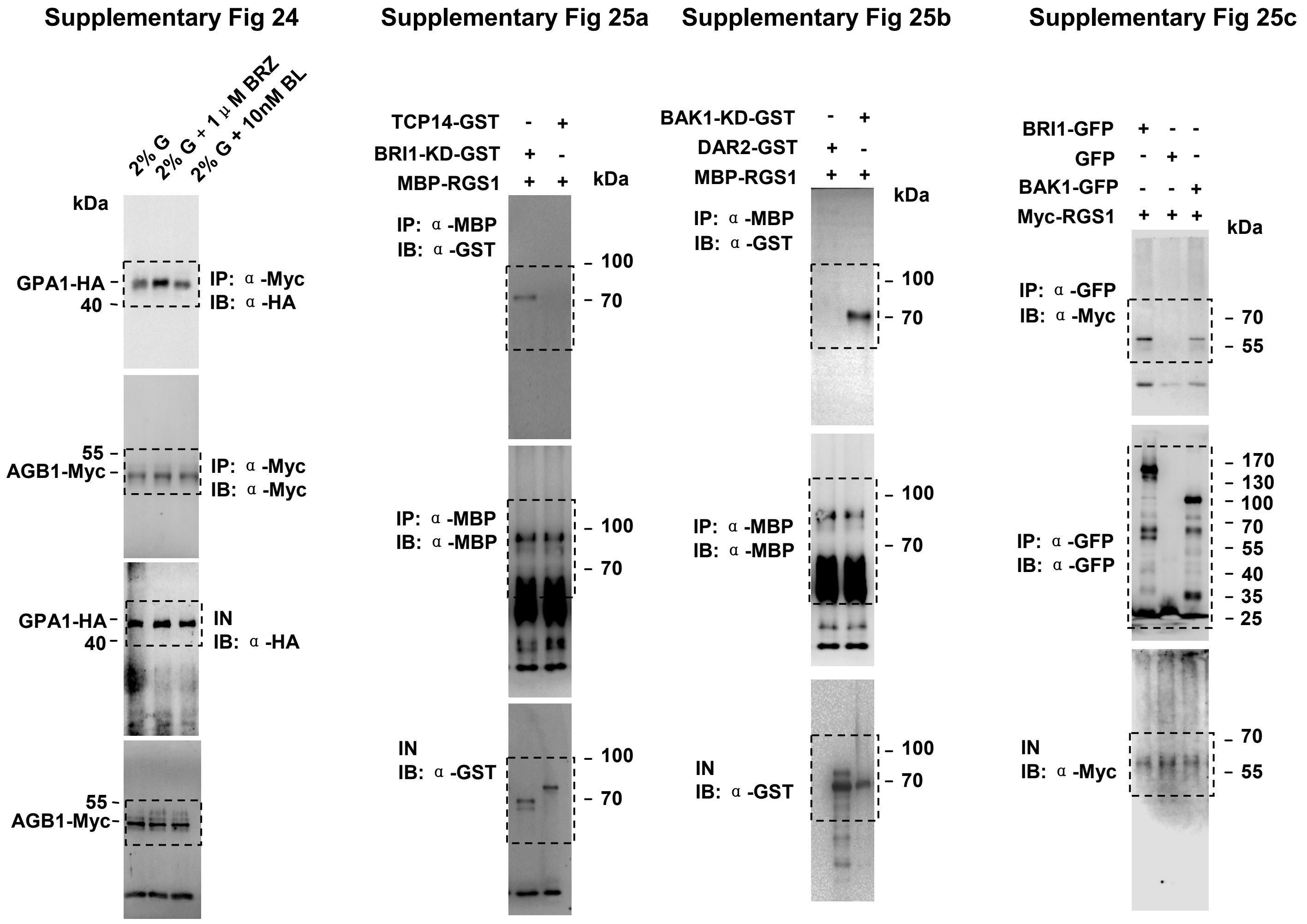

Supplementary Figure35 | Uncropped images of blots shown in Supplementary Fig. 24 and 25.

Supplemental Table 1. Phosphopeptides of AGB1

Sequence	phosphoRS Site Probabilities （%）	position
RLQLLDtDVAR	T(7): 100.0	T34
TRVsFGATDLVccR	S(4): 95.6	S49
HAVAtEtVNNLR	T(5): 100.0; T(7): 100.0	T14 T16
TFHGHEGDVNtVK	T(11): 100.0	T253
AVRtFHGHEGDVNTVK	T(4): 100.0;	T243
LIVWNALTSQKtHAIK	T(12): 93.2	T100
YSAAQGRtRVsFGATDLVccR	T(8): 96.2; S(11): 99.5	T46 S49
VSFGAtDLVccR	T(6): 100.0	T53
TFHGHEGDVNtVKFFPDGYR	T(11): 100.0;	T253
AVRtFHGHEGDVNtVKFFPDGYR	T(4): 100.0; T(14): 100.0	T243 T253
tFHGHEGDVNtVKFFPDGYR	T(1): 100.0; T(11): 100.0	T243 T253
HAVAtETVNNLR	T(5): 100.0;	T14
LQLLDTDVARYsAAQGR	S(12): 98.9	S40
RLQLLDTDVARYsAAQGR	S(13): 97.7	S40
LQLLDtDVARYsAAQGR	T(6): 100.0; S(12): 99.2	T34 S40
AVRtFHGHEGDVNtVK	T(4): 100.0; T(14): 100.0	T243 T253
FGtGSDDGTcR	T(3): 100.0	T265
IVsASQDGR	S(3): 100.0;	S82
HAVAtEtVNNLRDQLR	T(5): 100.0; T(7): 100.0	T14 T16
LQLLDtDVAR	T(6): 100.0	T34
VYsLDWTPER	S(3): 93.2	S70
TLQGHtGKVYSLDWTPER	T(6): 100.0;	T65
HAVAtETVNNLRDQLR	T(5): 90.8;	T14
tFHGHEGDVNtVK	T(1): 100.0; T(11): 100.0	T243 T253
ERHAVATEtVNNLRDQLR	T(7): 89.0	T14

Supplemental Table 2. Phosphopeptides of AGG3

Sequence	phosphoRS Site Probabilities （%）	position
EVSDFVVANsDPLIPAQR	S(10): 100.0	S92
ESAAGGVssSSLAPSSLPPPRPK	S(8): 98.4; S(9): 86.5	S21 S22
ESAAGGVSsSSLAPSSLPPPRPK	S(9): 84.9	S22
FIEGVQPAsR	S(9): 100.0	S78
ESAAGGVssSSLAPsSLPPPRPK	S(8): 98.6; S(9): 89.1	S21 S22
sPPEYPDLYGK	S(1): 100.0	S37

Supplemental Table 3. Primers used in this study

Name	Primers
**Primers for verifying T-DNA**	
SALK_115996LP	AGCTGTGGAGCTTGAATCTTG
SALK_115996RP	CTGAAGCTCATTACCACGAGC
SALK_061896LP	TCATTAGATTGGACACCGGAG
SALK_061896RP	TGTGAATCCTGCTGTAATCCC
SAIL_1209_B01LP	CACATGCACGAACACATTAGG
SAIL_1209_B01RP	CGAAAATGTCTGCTCCTTCTG
SALK_116202-LP	CATGACATCATCATCATTCGC
SALK_116202-RP	ATTTTGCAGTTTTGCCAACAC
SALK_LBa1	TGGTTCACGTAGTGGGCCATCG
SAIL_LB1	GCCTTTTCAGAAATGGATAAATAGCCTTGCTTCC
**Primers for constructs in pull-down assays**	
GST-BRI1-KD-FP	GGTTCCGCGTGGATCCCCGGCGATGGGTAGAGAGATGAGGAAGAG
GST-BRI1- KD-RP	ATGCGGCCGCTCGAGTCGATCATAATTTTCCTTCAGGAA
GST-bri1-301-KD-FP	GGTTCCGCGTGGATCCCCGGCGATGGGTAGAGAGATGAGGAAGAG
GST-bri1-301-KD-RP	ATGCGGCCGCTCGAGTCGATCATAATTTTCCTTCAGGAA
GST-BAK1-KD-FP	GGTTCCGCGTGGATCCCCGGCGatgCCTGCATCTCCACCGCCTCCTATCT
GST-BAK1-KD-RP	ATGCGGCCGCTCGAGTCGATTATCTTGGACCCGAGGGGT
MBP-GPA1-FP	ATCGAGGGAAGGATTTCAGAAATGGGCTTACTCTGCAGTAGA
MBP-GPA1-RP	GGCCAGTGCCAAGCTTGCCTGTAAAAGGCCAGCCTCCAGTA
MBP-AGB1-FP	ATCGAGGGAAGGATTTCAGAAATGTCTGTCTCCGAGCTCAA
MBP-AGB1-RP	GGCCAGTGCCAAGCTTGCCTGAATCACTCTCCTGTGTCCTC
MBP-AGG1-FP	ATCGAGGGAAGGATTTCAGAAATGCGAGAGGAAACTGTGGT
MBP-AGG1-RP	GGCCAGTGCCAAGCTTGCCTGAAGTATTAAGCATCTGCAGC
MBP-AGG2-FP	ATCGAGGGAAGGATTTCAGAAATGGAAGCGGGTAGCTCCAA
MBP-AGG2-RP	GGCCAGTGCCAAGCTTGCCTGAAGAATGGAGCAGCCACATC
MBP-AGG3-FP	ATCGAGGGAAGGATTTCAGAAATGTCTGCTCCTTCTGGCGG
MBP-AGG3-RP	GGCCAGTGCCAAGCTTGCCTGGAAAGCTAAACAACAAGGAT
MBP-RGS1-FP	ATCGAGGGAAGGATTTCAGAAATGGCGAGTGGATGTGCTCT
MBP-RGS1-RP	GGCCAGTGCCAAGCTTGCCTGACCGGGACTACTGCATCTGG
**Primers for constructs in Co-IP assays**	
Myc-AGB1-FP	GGATCCTAATGTCTGTCTCCGAGCTCAA
Myc-AGB1-RP	GAGCTCAAATCACTCTCCTGTGTCCTC
Myc-AGG3-FP	GGATCCTAATGTCTGCTCCTTCTGGCGG
Myc-AGG3-RP	GAGCTCAGAAAGCTAAACAACAAGGAT
Myc-RGS1-FP	GGTACCCATGGCGAGTGGATGTGCTCTAC
Myc-RGS1-RP	TACGTATTAACCGGGACTACTGCATCTG
**Primers for constructs in BIFC assays**	
BRI1-nYFP-FP	CAGGCCTGGCGCGCCACTAGTATGAAGACTTTTTCAAGCTTCTTTC
BRI1-nYFP-RP	CCCGGGAGCGGTACCCTCGAGTAATTTTCCTTCAGGAACTT
BAK1-nYFP-FP	CAGGCCTGGCGCGCCACTAGTATGGAACGAAGATTAATGATCCC

BAK1-nYFP-RP	CCCGGGAGCGGTACCCTCGAGTCTTGGACCCGAGGGGTATT
GPA1-cYFP-FP	CAGGCCTGGCGCGCCACTAGTATGGGCTTACTCTGCAGTAGA
GPA1-cYFP-RP	CCCGGGAGCGGTACCCTCGAGTAAAAGGCCAGCCTCCAGTA
AGB1-cYFP-FP	CAGGCCTGGCGCGCCACTAGTATGTCTGTCTCCGAGCTCAA
AGB1-cYFP-RP	CCCGGGAGCGGTACCCTCGAGAATCACTCTCCTGTGTCCTC
AGG3-cYFP-FP	CAGGCCTGGCGCGCCACTAGTATGTCTGCTCCTTCTGGCGG
AGG3-cYFP-RP	CCCGGGAGCGGTACCCTCGAGGAAAGCTAAACAACAAGGAT
**Primers for constructs in vivo phosphorylation assays**	
GFP-AGB1-FP	ATGTCTGTCTCCGAGCTCAAAG
GFP-AGB1-RP	AATCACTCTCCTGTGTCCTCCA
**Primers for constructs in plant transformation**	
gBAK1-GFP-pro-FP	TGCATGCCTGCAGGTCGATTATCAATGTGTTAAAGAATTC
gBAK1-GFP-pro-RP	TTTATCCTCAAGAGATTAAA
gBAK1-GFP-CDS-FP	TTAATCTCTTGAGGATAAAATGGAACGAAGATTAATGATCCC
gBAK1-GFP-CDS-RP	TACCGGGCCCCCCCTCGATCTTGGACCCGAGGGGTATT
**Primers for site-directed mutagenesis**	
AGB1-T14A-FP	CGCCGTCGCTgCGGAGACCGT
AGB1-T14A-RP	TGGCGTTCTTTGAGCTCGGAGAC
AGB1-S40A-FP	GGCGAGGTATgCAGCGGCGCA
AGB1-S40A-RP	ACATCGGTATCGAGGAGCTGGAG
AGB1-T243A-FP	AGCAGTGCGTgCCTTTCATGG
AGB1-T243A-RP	CGGCTTGCAGCACGAGTG
AGB1-T243A T253A-FP	agggagatgttaatgCGGTCAAGTTCTTTCCGGATGG
AGB1-T243A T253A-RP	cgtgaccatgaaaggcACGCACTGCTCGGCTTGC
AGB1-T14D-FP	CGCCGTCGCTgatGAGACCGT
AGB1-T14D-RP	TGGCGTTCTTTGAGCTCGGAGAC
AGB1-S40D-FP	GGCGAGGTATgatGCGGCGCA
AGB1-S40D-RP	ACATCGGTATCGAGGAGCTGGAG
AGB1-T243D-FP	AGCAGTGCGTgaCTTTCATGG
AGB1-T243D-RP	CGGCTTGCAGCACGAGTG
AGB1-T243D T253D-FP	agggagatgttaatgatGTCAAGTTCTTTCCGGATGG
AGB1-T243D T253D-RP	cgtgaccatgaaagtcACGCACTGCTCGGCTTGC
AGG3-S21A-FP	TGGTGGAGTGgcTTCATCGTCTCTTG
AGG3-S21A-RP	GCAGCTGATTCTTTTCCTC
AGG3-S21A S22A-FP	TGGTGGAGTGgctgCATCGTCTCTTGC
AGG3-S21A S22A-RP	GCAGCTGATTCTTTTCCTC
AGG3-S21D-FP	TGGTGGAGTGgaTTCATCGTCTCTTG
AGG3-S21D-RP	GCAGCTGATTCTTTTCCTC
AGG3-S21D S22D-FP	TGGTGAGTGgatgatTCGTCTCTTGC
AGG3-S21D S22D-RP	GCAGCTGATTCTTTTCCTC
AGG3-T143A-FP	CTGCAACTGTgCATCTTGCAG
AGG3-T143A-RP	CACTTGGGTTTCCTCAGATG
AGG3-T143D-FP	CTGCAACTGTgacTCTTGCAG
AGG3-T143D-RP	CACTTGGGTTTCCTCAGATG

AGG3-T199A-FP	TTGCAGTTGCgCTCGACCGTC
AGG3-T199A-RP	CTGCGGAAACAGCTGGGAAT
AGG3-T199D-FP	TTGCAGTTGCgaTCGACCGTC
AGG3-T199D-RP	CTGCGGAAACAGCTGGGAAT
